# Clinical decision-making and adaptive expertise in residency: a think-aloud study

**DOI:** 10.1186/s12909-022-03990-8

**Published:** 2023-01-12

**Authors:** Maria Louise Gamborg, Mimi Mehlsen, Charlotte Paltved, Sigrid Strunge Vetter, Peter Musaeus

**Affiliations:** 1 Centre for Educational Development, Aarhus University, Aarhus C, Denmark; 2 Coporate HR MidtSim & Department of Clinical Medicine, Faculty of Health, Aarhus University, Central Denmark Region, Palle Juul-Jensens Boulevard 82, DK-8200 Aarhus N, Denmark; 3 Department of Psychology, Faculty of Business and Social Sciences, Aarhus University, Bartholins Allé 11, 8000 Aarhus C, Denmark

**Keywords:** Clinical decision-making, Adaptive expertise, Residents, Elderly patients, Emergency medicine

## Abstract

Clinical decision-making (CDM) is the ability to make clinical choices based on the knowledge and information available to the physician. It often refers to individual cognitive processes that becomes more dependent with the acquisition of experience and knowledge. Previous research has used dual-process theory to explain the cognitive processes involved in how physicians acquire experiences that help them develop CDM. However, less is known about how CDM is shaped by the physicians’ situated cognition in the clinical environment. This is especially challenging for novice physicians, as they need to be adaptive to compensate for the lack of experience. The adaptive expert framework has been used to explain how novice physicians learn, but it has not yet been explored, how adaptive expertise is linked to clinical decision-making amongst novice physicians.

This study aimed to analyse how residents utilize and develop adaptive expert cognition in a natural setting. By describing cognitive processes through verbalization of thought processes, we sought to explore their CDM strategies considering the adaptive expert framework.

We used concurrent and retrospective think-aloud interviews in a natural setting of an emergency department (ED) at a university hospital, to query residents about their reasoning during a patient encounter. We analysed data using protocol analysis to map cognitive strategies from these verbalizations. Subsequently in a narrative analysis, we compared these strategies with the literature on adaptive expertise.

Fourteen interviews were audio recorded over the course for 17 h of observation. We coded 78 informational concepts and 46 cognitive processes. The narrative analysis demonstrated how epistemic distance was prevalent in the initial CDM process and self-regulating processes occurred during hypothesis testing. However, residents who too quickly moved on to hypothesis testing tended to have to redirect their hypothesis more often, and thus be more laborious in their CDM. Uncertainty affected physicians’ CDM when they did not reconcile their professional role with being allowed to be uncertain. This allowance is an important feature of orientation to new knowledge as it facilitates the evaluation of what the physician does not know.

For the resident to learn to act as an adaptive decision-maker, she relied on contextual support. The professional role was crucial in decisional competency. This supports current literature, which argues that role clarification helps decisional competency. This study adds that promoting professional development by tolerating uncertainty may improve adaptive decisional competency.

**Supplementary Information:**

The online version contains supplementary material available at 10.1186/s12909-022-03990-8.

## Background

Clinical decision-making refers to the ability to process information and make decisions on diagnosis and treatment for patients. Thus, clinical decision-making or clinical reasoning can be conceived as an hypothetico-deductive aptitude and pattern-recognizing competency [1–3]. Residents’ learning of clinical decision-making is wrought with challenges due to the fundamental variability in the clinical environment coupled with residents’ scant knowledge and experience [4–11]. Furthermore, it is well-known that dual-process clinical decision-making is prone to be influenced by biases and heuristics [12]. Schwartz [6] argued that it was problematic to translate decision science with a focus on heuristics and biases to medical problems. This lack of a translational approach to clinical decision-making, he argued, has led to a discrepancy between the basic scientific study of reasoning versus every day, clinical decision-making [6]. Schwartz [3] argued that a processual account was necessary and that medical decision science thus needed both basic science (cognitive and ethnographic, etc.) research on decision-making as well as applied medical educational research on how to relate this to individual decisional competencies embedded in societal structures.

These challenges have sparked several initiatives for residency training on how to support every-day clinical decision-making, such as technological educational aids [13], reflective interventions [14], and bias-reduction aids and strategies [8, 15–19]. However, there is no clear evidence that decision aids such as algorithms outperform unaided decision-making [20] or that de-biasing strategies reduce errors [19, 21, 22]. Furthermore, junior doctors’ decisions might be largely motivated by senior staff’s decisions rather than independent decisional competency [5].

### The adaptive expert framework

An alternative concept of developing competence in clinical decision-making can be gleaned through Hatano’s [23] framework of adaptive expertise. This framework conceptualizes the acquisition of expert decision competency through a cognitive model of procedural and conceptual knowledge [23, 24]. Procedural knowledge refers to know-how or knowledge of what to do. Conceptual knowledge on the other hand, refers to a grasp of medical notions and explanations about why a given action is performed. Thus, the physician needs to form mental models of procedures in line with this knowledge. The adaptive expert can represent these models in flexible ways that evolves when unknown situations arise. Here, adaptive experts can identify when their current understanding of the procedure is inadequate. They can adjust their representations while solving the problem at hand and this adaptive strategy serves to hone their competency. As a result, upon critically reflecting on how this new procedural knowledge fits into the larger concept, the adaptive expert expand their conceptual understanding of both routine and challenging procedures, as a continuous evaluation of their actions [25, 26]. Adaptive experts balance these routine and adaptive practices in what Schwartz et al. (27;P37) called an ‘optimal corridor of adaptability’ indicating that the progression of professional development is spatial (the metaphor of a corridor) and must take advantage of the right moment. This occurs through appropriately applying both new conceptual knowledge, innovative solutions, and efficient automatic or routinized practices [27, 28].

To identify their knowledge gaps, physicians need to learn to position themselves as observers of their knowledge as they apply procedural and conceptual knowledge. This is called *epistemic distance* [29] and refers to the ability to flexibly monitor the adequacy of one’s knowledge to solve a problem, construct deeper understandings, and develop new solutions. During this process, the expert physician *self-regulates* towards closing these gaps in knowledge, by applying metacognition and critical thinking [28]. At the same time, physicians are *oriented towards new knowledge* and actively seeks out learning opportunities [29–31]. This competency reflects a fundamental attitude that guides the physician in her professional development [32]. It is associated with how she authors or construes her professional identity, i.e. whom she takes herself to be and is taken to be as a becoming physician [29].

### Adaptive expertise in residency and medical education

Residency training involves a myriad of learning situations and teachers [33]. Therefore, medical educators have highlighted adaptive expertise as an important competency to learn in residency [34]. Research has emphasized that adaptive expertise is a social and relational phenomenon where social and material bonds are formed, negotiated, and reshaped constantly [35]. Thus, novices might unreflectively mimic routine practices, if these practices are not explicitly reinstated [36]. Such awareness of residents might potentially affect patient safety [11].

Experts are characterised by their aptitude in forming meaningful patterns of information with organized structures of knowledge that reflect a situational variation. Experts’ rapid and flexible retrieval of situationally relevant knowledge is what defines experts and make them reliable decision-makers [37, 38]. While residents may acknowledge that variability in medical practice requires more than routine knowledge, studies on their conceptual understanding of expertise has been shown to reflect a routine approach to developing expertise [39], which may influence their approach to learning and ability to learn adaptive expertise.

The framework of adaptive expertise is a fruitful way of researching decisional competency [40]. Furthermore, is helps design training and thus might prepare residents for future learning [41]. Yet, only limited research exists that explore the development of adaptive expertise within resident physicians [32]. Empirical research on adaptive expertise has mostly focused on socio-cultural elements or individual predispositions. These are thought to mediate the development of adaptive expertise. However, in medical education little empirical research has focused on studying cognitive processes ecologically in the wild [42, 43]. This could entail documenting cognitive thought processes to build transactional models that takes the mutuality of physician and clinical environment into account. In particular, there is a lack of research on how the real-life thought processes of residents unfold in clinical practice [25] and how these processes are mirrored by the adaptive processes between expert and clinical environment. This might lead the way to a better understanding of early-career interventions which can, in turn, promote better clinical decision-making.

### Diagnostic reasoning

The physician in the ED often operates as an agent responsible for intake and sorting of patients. Thus, diagnosis is a determining action that regulates the flow of patients. Furthermore, it is time-dependent and regulates whether the patient is treated immediately, sent to another department or discharged. These clinical decisions are based on the physician’s working hypothesis and in the Danish EDs, residents are formulating the preliminary working hypothesis. Researchers have conceived of diagnosis as an iterative problem-solving process of testing hypotheses, and collecting, categorising, and matching data [44]. This view of diagnostic reasoning is much in line with being epistemically aware and self-regulation processes in the framework of adaptive expertise [28, 45]. However, none has investigated how these coincide. Thus, the research focus in this study were cognitive processes leading up to diagnosis. This focus was chosen to sequence the problem-solving diagnostic process and investigate the temporal distribution of adaptive expert cognition.

## Methods

### Design

We used a think-aloud interview method to provide insight into informants’ cognitive processes [46, 47]. The think-aloud method aims to describe a person’s thought processes as they happen [46, 48]. Two interview methods were used with each informant. First, we used a concurrent think-aloud interview to record residents’ verbalization of information in their working memory during the diagnostic process [47]. Second, we used a retrospective interview to explore residents’ rationales for their decision-making [47]. The combination of these two methods has been used in other studies to provide a comprehensive description of thought processes [49, 50]. As the concurrent method in natural setting allows for rich and accurate descriptions, the retrospective recall allows for contextualizing and detailing those descriptions [49, 50]. It has been argued that natural settings provide a realistic testbed for CDM [50, 51]. For this reason, we used the concurrent interview during real patient encounters. Transcripts of these encounters then provided case material for the retrospective think-aloud. Additionally, the primary investigator, MLG, performed observer participant observations [52] before, during, and after the concurrent think-aloud interviews.

### Setting and case of geriatric emergency medicine

Inclusion criteria for patient encounters were patients over the age of 60, admitted to the emergency department. It has been argued that complex cases provide the best opportunity to explore CDM [53]. Geriatric emergency cases are clinically complex and they were selected because residents report lower confidence treating geriatric patients, compared to other adult patient groups [7, 9]. Specifically elderly patients are complex due to comorbidity, social factors, and communication issues [7, 54]. Furthermore the emergency setting is highly unpredictable [55]. Thus, residents need to develop adaptive expertise to handle the unpredictable and demanding setting of the emergency department [34].

### Informants

Ten volunteering residents were recruited through a chief physician from the Emergency Department (ED) at one university hospital. We aimed to recruit residents in their first postgraduate year (PGY1). In all, seven PGY-1 residents were included in the final dataset. Two residents were excluded from the dataset due to scheduling issues related to Covid-19 restrictions and one resident was excluded after the concurrent think-aloud interview, as it was not possible to perform the retrospective interview think-aloud interview.

### Procedure

The first author (MLG) conducted all interviews. Recruitment of residents took place at their first day of their introduction. Here, they were introduced to the think-aloud method. In this introduction, a brief presentation of the goal and procedure of the method was given, as well as instruction on how they should verbalize their thoughts both during the concurrent (what they were thinking) and retrospective (why they thought so) interviews. They engaged in a short practice session on the day of the concurrent think-aloud interview. Here, they would think-aloud on their preparation for another patient, where MLG would instruct informants in verbalizing second-order thoughts [46, 47, 56], correct them if they were describing rationales instead, and answer any questions they had, regarding the method of verbalization. This method was chosen, in opposition to Ericsson and Simon's [47] method of using a mathematical problem, as it provided more natural verbalization, and tapped into their habit of teaching medical students, easing them into the method more naturally and efficiently. During the concurrent interview, MLG paid close attention to the types of verbalizations used by physicians. When needed, she reminded and guided the informants to describe what they were thinking concretely, rather than their rationales or abstract ideas about why they were doing something. All staff in the ED were informed of the study before data collection and were given information about data collection including notification when they were being audio recorded.

### Ethical considerations

This study was approved by the Danish Data Inspectorate (j.2016–051-000,001, case 1487), and was exempted from ethical approval from the Central Denmark Region Committees on Research Ethics (j.1–10-72–1-19). During recruitment, all informants were provided with oral and written information about the project. Written informed consent was obtained from all informants when recruited. Verbal informed consent to partake in the study was obtained from all staff interacting with the informant, as well as all patients treated by them, and their relatives. Patients were informed that no personal information was obtained and that the transcriptions would not include any identifiable information.

#### Concurrent think-aloud interview

The concurrent interviews were audio-recorded during the geriatric patient encounter. The interviewer recorded second-order verbalizations of thoughts in line with previous studies [46, 47, 56], by probing the resident to describe *what* they were thinking rather than *why* they were thinking it both before, during, and after the patient encounter. Initially after interacting with the patient, MLG would probe for any thoughts they were not able to speak in front of the patient. Audio recording started when the resident prepared for the patient meeting. The recording would only be paused in case the resident had to take care of another patient or during long waiting periods. The audio recording was also stopped when the diagnosis and treatment plan was decided on.

#### Retrospective think-aloud interview

The recordings were transcribed ad verbatim. This provided a case description for the subsequent retrospective think-aloud interview. The average time between interviews was 16 days (ranging from four to 44 days). At the beginning of the interview, residents were instructed to think back on the patient encounter and explain what they could recall from the interaction. They were also asked if it had led to any further reflections or if they had followed up on the patient, afterward. They were then instructed to read through the transcript and use the same method of thinking aloud as in the concurrent interview, verbalizing their immediate thoughts. Additionally, they were carefully instructed in also verbalising their rationales (why they did a specific action or had a specific thought). Furthermore, they were instructed to add any thoughts they were unable to verbalize in the clinical setting, out of consideration to other staff or the patient.

#### Observations

Using focused ethnography [57] MLG performed participant observations [52] of behaviours and social interactions mainly between physicians and patients. This approach also involved studying physicians’ use of artefacts in diagnostic reasoning before, during, and after the patient encounter. Field notes were written into a word document immediately after, to provide greater detail by fleshing out the field notes. These were then added in the chronologically appropriate places in the transcript for the retrospective interview. During analysis and reporting, the field notes provided rich descriptions of the environment, interactions, and procedures that the residents engaged in.

### Data analysis

The analysis was two-fold. Firstly, protocol analysis aimed to identify what information residents identified, and which cognitive processes they used to do so. Secondly, we performed a narrative analysis of these informational concepts and cognitive processes, to illustrate and describe the diagnostic processes of residents, in relation to the adaptive expert framework.

#### Protocol analysis

Protocol analysis is a well-researched method of analysis, which can be applied to think-aloud verbal data of complex decision-making in clinical settings [49]. We merged the two transcripts from the concurrent and retrospective think-aloud interview into one verbal protocol for each resident, which underwent protocol analysis as described by Ericsson and Simon [56]. We conducted referring phrase analysis and script analysis in line with previous studies [58–60]. In the referring phrase analysis, we identified the types of information (referred to as information concepts) used in CDM from the noun or noun-concepts in the sentences. In the script analysis, we analysed how this information was used as cognitive operators for CDM (referred to as cognitive processes). This analysis was summarized, allowing us to describe the type of information as well as the cognitive processes used during clinical diagnostic processes.

#### Narrative analysis

The narrative analysis was performed on both observational and transcript data. We collapsed the cognitive processes identified in the script analysis into a six-step decision-making process, based on research on diagnostic reasoning, which describe information gathering, hypothesis generation, and testing [19, 61–65]. We added the processes of confirmation and diagnosing, as they were identified in the script analysis. After a comparison of the scripts, the authors reached consensus, that these were separate processes. This six-step diagnostic decision-making process is described in more detail below:

1. Gathering information: verbalisations that demonstrated specific actions to gather information about the patient.
2. Generating hypothesis: verbalizations that reflected which hypothesis the resident was investigating and what their working hypothesis was.
3. Identifying cues: verbalizations that reflected which specific information the resident acted on and noticed regarding the working hypothesis.
4. Testing hypotheses: verbalisations that demonstrated concrete actions taken towards confirming their working hypothesis.
5. Confirming hypotheses: verbalisations that indicated that the resident was confirmed in their working hypothesis.
6. Diagnosing: verbalisations that demonstrated a tentative conclusion to the diagnostic process, iterating the confirmed diagnosis to be acted on regarding treatment planning.

Using the verbal protocols, we sequenced the entire patient encounter based on these six steps, for each informant. We visualised these sequences in figures, which allowed us to illustrate the diagnostic process and provide rich case descriptions of each informant. Furthermore, by using the framework of adaptive expertise [28, 29, 40, 45] as a sensitising concept, we identified and juxtaposed the adaptive cognitive tasks to the diagnostic process. This was done to evaluate when and how adaptive practices were employed by residents during diagnostic reasoning processes. This deductive and inductive analysis was performed by the main author (MLG), analysing all raw data into the adaptive expert framework and then overlaying this data with the six-step diagnostic process.

### Methodological rigour and reflexivity

The research team was comprised of three researchers in medical education, three with a Ph. D. in psychology (MLG, MM, and PM), one student (SS), and one with a background in medicine (CP). Everyone but the student helper had extensive experience with qualitative and quantitative research methods. Furthermore, MLG and MM had prior practical experience with geriatric psychiatry. Thus, the team consisted of an experienced group with knowledge of methodology, educational theories, and medical practice for this study. This mix of methodological experience and background in clinical psychology, and clinical medicine influenced how we formulated the research question. It led us to ground our approach in a post-positivist stance [66, 67], aiming to describe clinical decision-making as an interplay between theories, hypotheses, knowledge, and values of the residents and their environment. Additionally, we as researchers naturally influenced both data collection and analysis. The focus on geriatric patients sprung from professional experience from the primary investigator (MLG), who had prior work experience with elderly patients and EDs as a clinical psychologist in geriatric psychiatry. This facilitated data collection as she understood relevant medical terminology related to geriatric patients. But it also made MLG more than a passive observer. For instance, when communicating this professional experience to the informants, one of them asked for her professional opinion. She gave her insight and noted this in the audit trail, for an analysis of a possible information bias during analysis. However, the content and process of the decision-making processes were markedly different from her personal experience as became apparent during the protocol analysis.

In the protocol analysis, the first author (MLG) collected all data and conducted the primary analysis under supervision from the other authors who were chartered psychologists (MM and PM) and a senior physician with expert knowledge of emergency medicine (CP). Samples of data were analysed by a co-author (PM) and a student helper (SS) to discuss and reach consensus on information concepts and cognitive processes. This analysis was then discussed with another co-author (MM).

In the narrative analysis, sequencing the diagnostic process and deductively identifying adaptive cognition MLG performed the initial analysis. Then, all co-authors reviewed the analysis to reach consensus on sequencing, and interpretations of adaptive practices, and review the mapping to the diagnostic process. The author group would discuss findings, continually, during analysis.

The intention of collecting both concurrent and retrospective think-aloud verbal data, served three purposes: to allow second and third order thoughts, to mitigate ethical issues regarding verbalization, and to increase trustworthiness, by triangulation of different data sources, and several means of member-checking [68]. In terms of rigorousness [69], the combination of verbal and observed data provided an in-depth and rich description of informants’ cognitive processes, allowing for adequate data for the narrative analysis. Furthermore, the concurrent verbalisation and direct observations added authenticity to the data, which was further supported by member-checking in the retrospective interviews which allowed cross-checking with the informant [50]. Thus, the analytic steps were appropriately and systematically taken and followed norms stated in the literature [47, 68]. This increases the trustworthiness of the findings [69]. In addition, by engaging in conversations after retrospective interviews on the ongoing and preliminary analysis of their concurrent interviews, informants found that they could relate to the initial findings. Several informants expressed surprise at the richness and detail of the transcripts, finding it easier to recall the intricacies of the individual patient encounter with this type of fine granularity of the records [69]. Finally, the ongoing analysis was discussed with the chief physician. This served as a member-checking technique, which ensured resonance between lived experiences and clinical practice as viewed from within the community [69].

## Results

### Informant characteristics

The final sample consisted of seven PGY-1 residents (4 female and 3 male) with an average of 2.5 months of experience at the time of their first interview, ranging from 48 to 111 days, after their graduation.

### Data set

Data consisted of 14 interviews, ranging from one to four hours, with the seven informants and 17 h of observation of patient-resident encounters. The interviews focused on one patient encounter for each informant resident.

### Protocol analysis

As described in the data analysis, protocol analysis consisted of two steps: 1) referral phrase analysis, and 2) script analysis [58].

#### Referral phrase analysis

By isolating the noun and noun sentences in the transcripts we were able to identify what kind of information residents verbalized during their diagnostic process. From this referral phrase analysis, 78 concepts of information were identified, where the most common are described in Table 1.

**Table 1:** Informational concepts identified in the referral phrase analysis

Concept	Definition	Verbal data
Actions	Statements related to their behaviour	The nurse has just finished her handover of the patient to Daniel: *“Yes, but let’s go in right away.”*
Patient characteristics	Patient characteristics with a consequence for the diagnostic process (e.g., gender or known pathology)	Casper is treating a fallen patient who reportedly suffers from dementia. During his preparation, Casper explains: *“Often, we are more generous with x-rays when they [patients] have dementia because the physical examination can be difficult.”*
Anamnesis	Patient history and description of events leading up to admission	Ellen is gaining an overview of the patient and explains while reading the electronic patient journal that the patient *“…has been here for outpatient control, where she was [treated] for a distal radius fracture.”*
Peer opinion	Opinions from other physicians, both residents and experienced	Julie has taken over a complex patient from another resident. She has conferred the patient with the available supervising physician who is an orthopaedic surgeon. But she is still unsure of the medical side of the problem. She remarks, *“… it’s good to talk to the tending emergency physician, as they are more attuned to the medical challenges…”*
Plan	Recounting the plan for receiving and treating the patient	Mark is treating a patient who has fallen and is in severe pain. He stops the physical examination and explains to the patient that as she is in so much pain, *“…we will do an x-ray first, and then I can examine you further if there’s no visible fracture.”*
Sign	Visible symptoms or test results related to the present diagnosis or patient state	Christina is examining a patient with suspected fracture on the ankle. She compares the sizes of the patients’ ankles and notes, *“There’s a visible swelling here.”*
Value	The meaning of symptoms to the diagnosis	During the handover from the EMTs, Mark retrospectively explains that some of the reported values puzzle him. He reflects that this affects his decision-making: *“I start to consider… because you shouldn’t receive a random patient with a heart rate of 35.”*
Referral	The referral notes are written by the referring physician before admission	Anne has been notified that there is a patient incoming, and remarks that *“…I will look him up… [And] see if there’s a referral note…”*

All names are pseudonyms

#### Script analysis

From this identification of what kind of information residents verbalized, we were able to infer how this information was used during the diagnostic process, indicating the cognitive processes employed by residents. From this script analysis we identified, in all, 46 cognitive processes among all residents. The cognitive processes that were most commonly identified are described in Table 2.

**Table 2:** Cognitive processes identified in the script analysis

Cognitive process	Definition	Verbal data
Choosing	Choosing between different options (i.e., examination or treatment)	Christina is examining a patient who may have a fracture to the ankle and is considering if it warrants an x-ray. She has acknowledged a significant swelling but decides, *“I actually think we should see if you can stand on it.”*
Concluding	Summarising and concluding on rationales for choices made	Ellen has been through the patient’s electronic journal and summarize her reflections: *“We definitely need to take an ECG.”*
Information seeking	Actively seeking out ways for gaining more information	The EMTs has just finished the handover to Mark, where an unusual heart rate is reported. He explains therefore that *“…the first I think I will do, is to take her hand and feel for her pulse…”*
Studying	Investigating information further, seeking a better understanding to make a diagnostic decision	Anne is examining a patient who has cut himself and explains that she usually repeats the patient’s answers, *“…so they can elaborate on their story a bit.”*

All names are pseudonyms

An additional table file contains a complete list of the 78 informational concept and 46 cognitive process codes [See Additional file 1].

We identified several commonalities among cognitive processes including choosing, concluding, information seeking, and studying. All residents utilized adaptive expert cognition through similar strategies. For instance, epistemic distance defined as monitoring level of knowledge and identifying knowledge gaps [29] was observed among all informants.

### Residents’ diagnostic reasoning

**Fig. 1:**
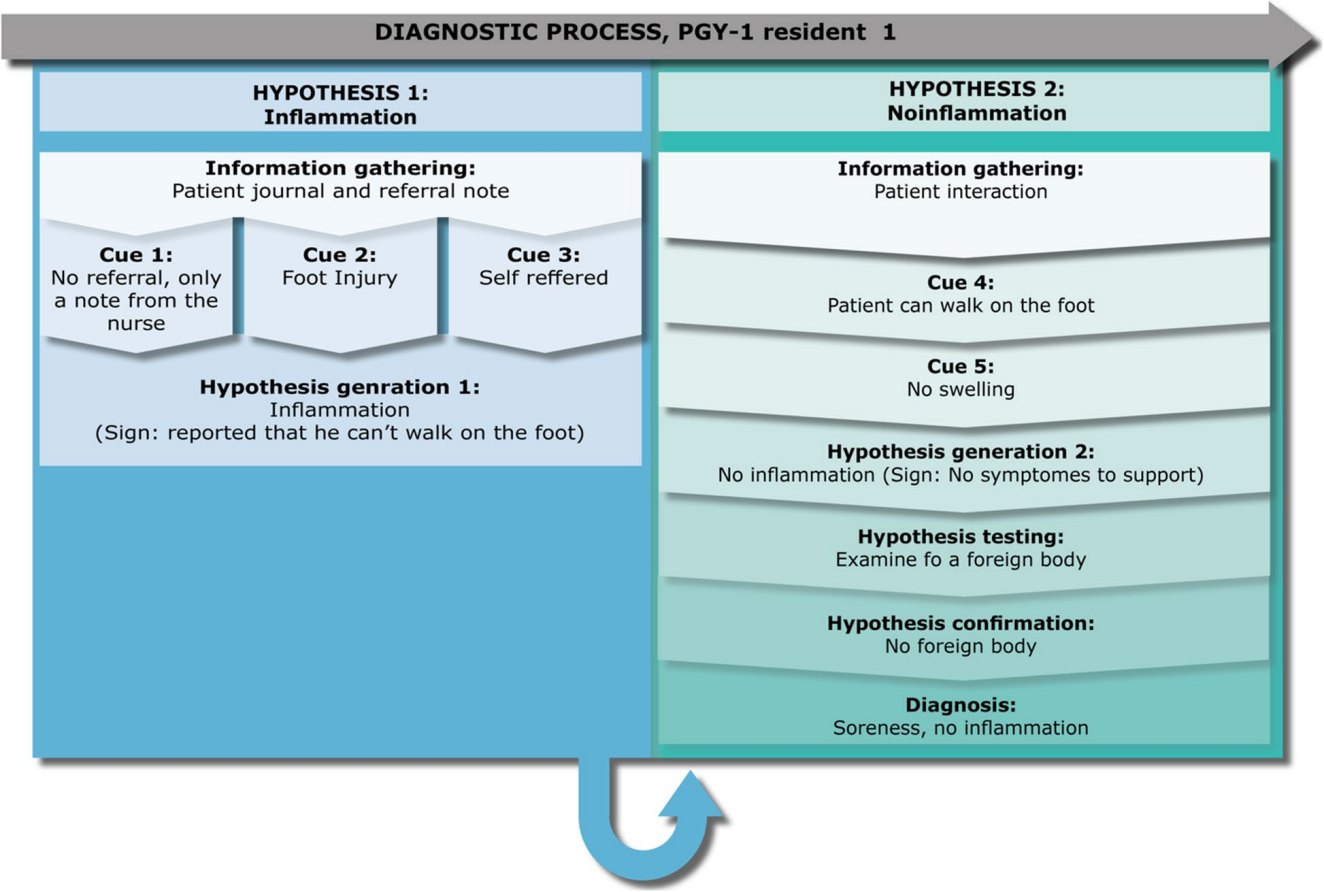
PGY-1 resident 1, “Anne” – a case of inflammation

**Fig. 2:**
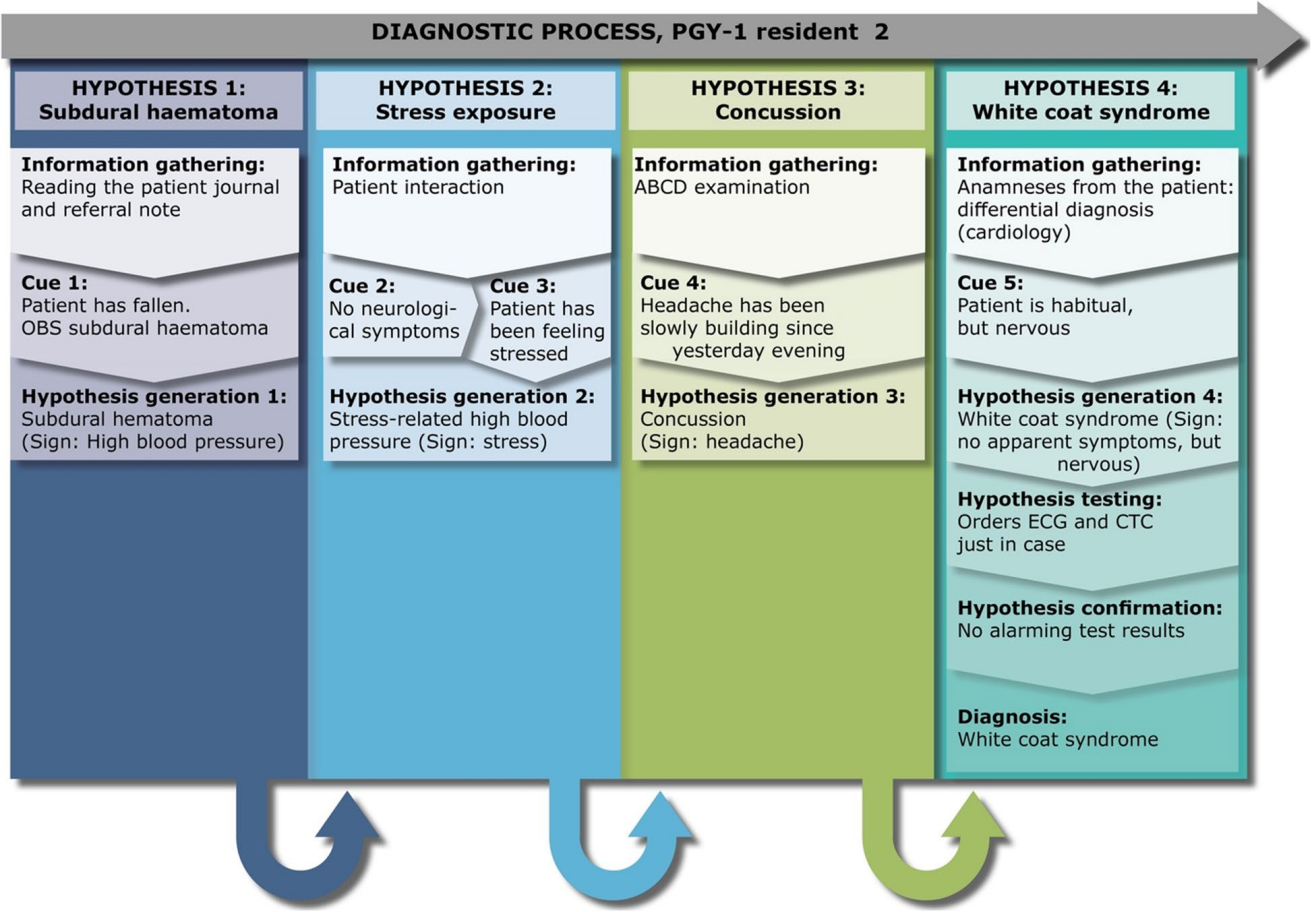
PGY-1 resident 2, “Ellen” – a case of high blood pressure

**Fig. 3:**
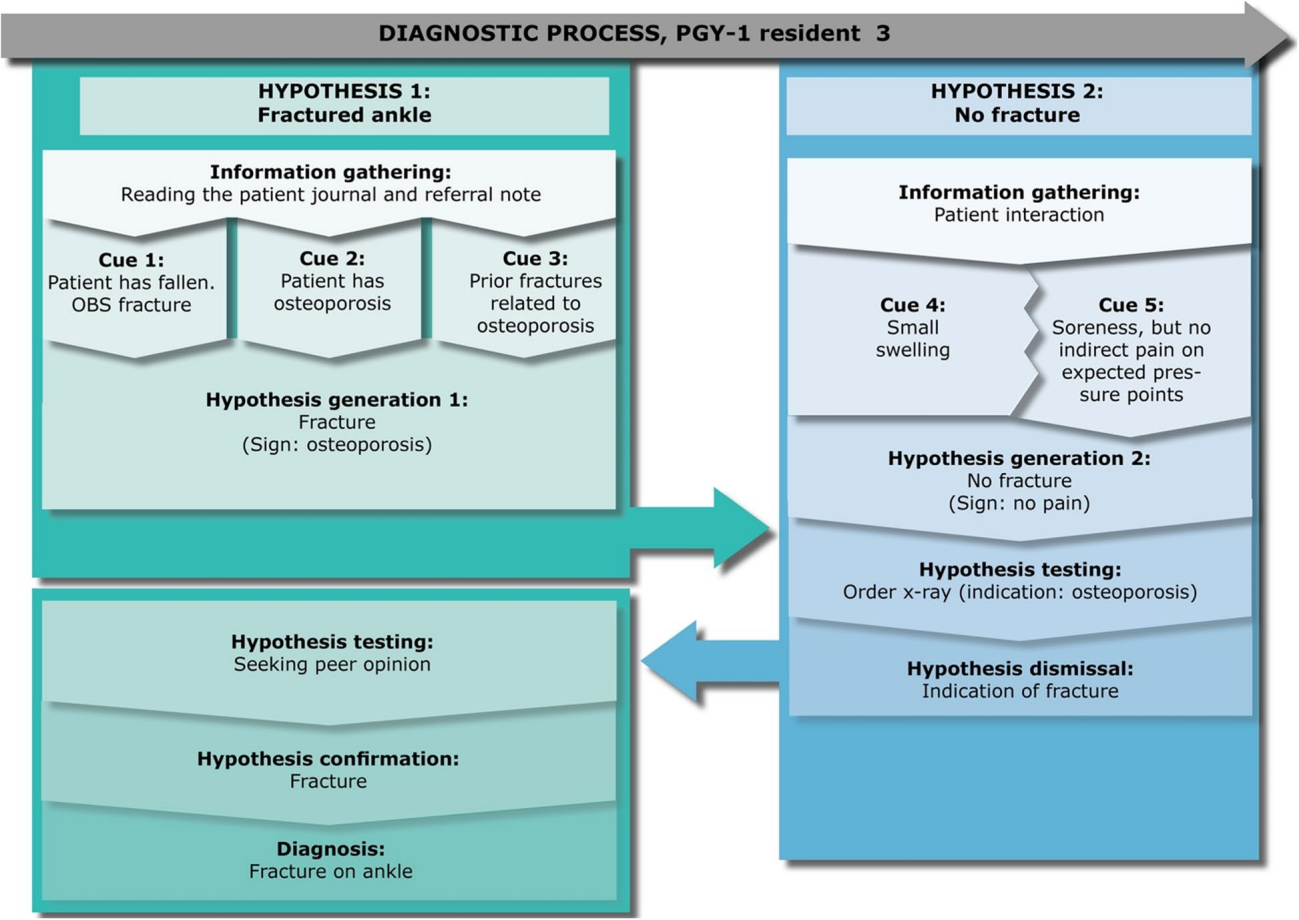
PGY-1 resident 3, “Christina” – a case of a fractured ankle

**Fig. 4:**
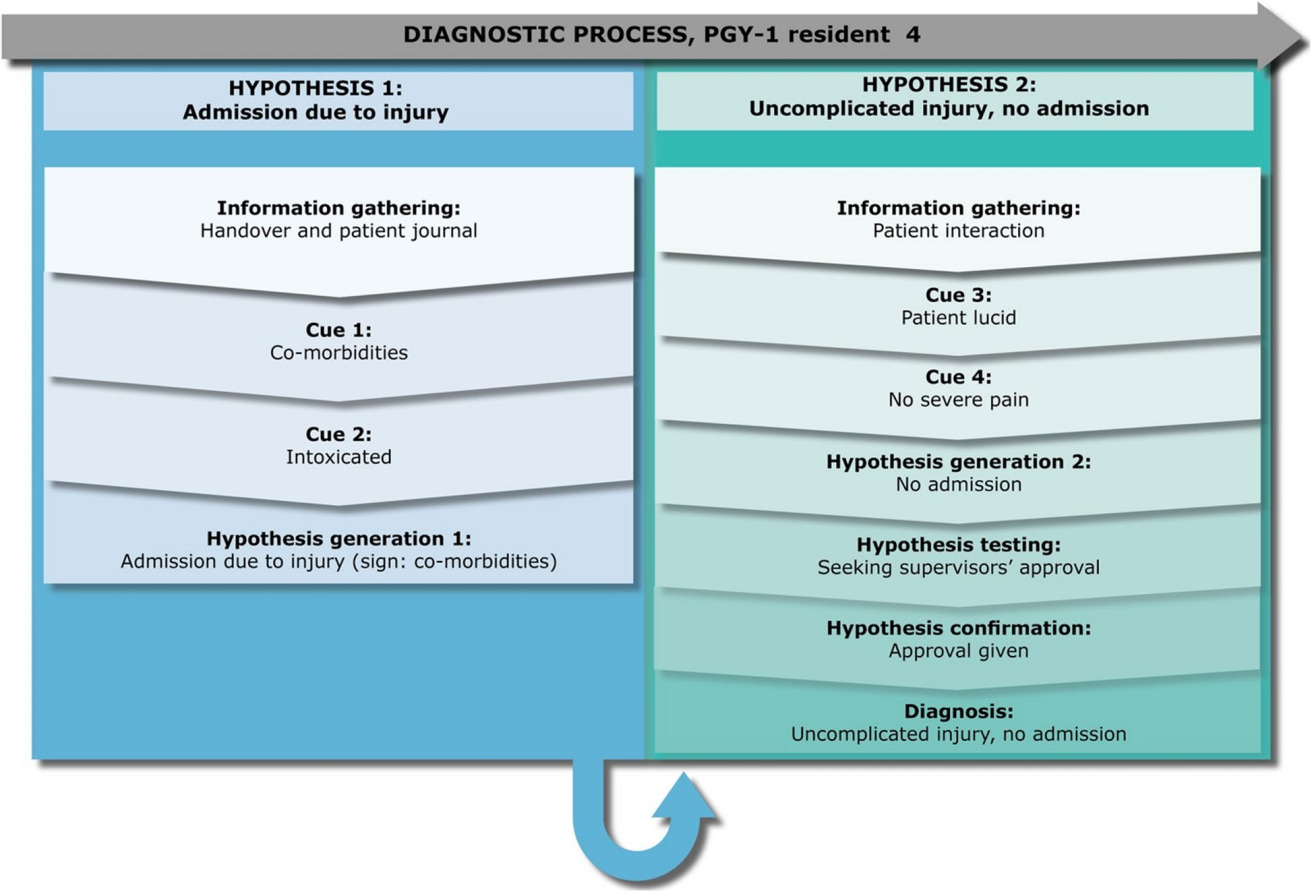
PGY-1 resident 4 “Julie” – a case of traumatic injury

**Fig. 5:**
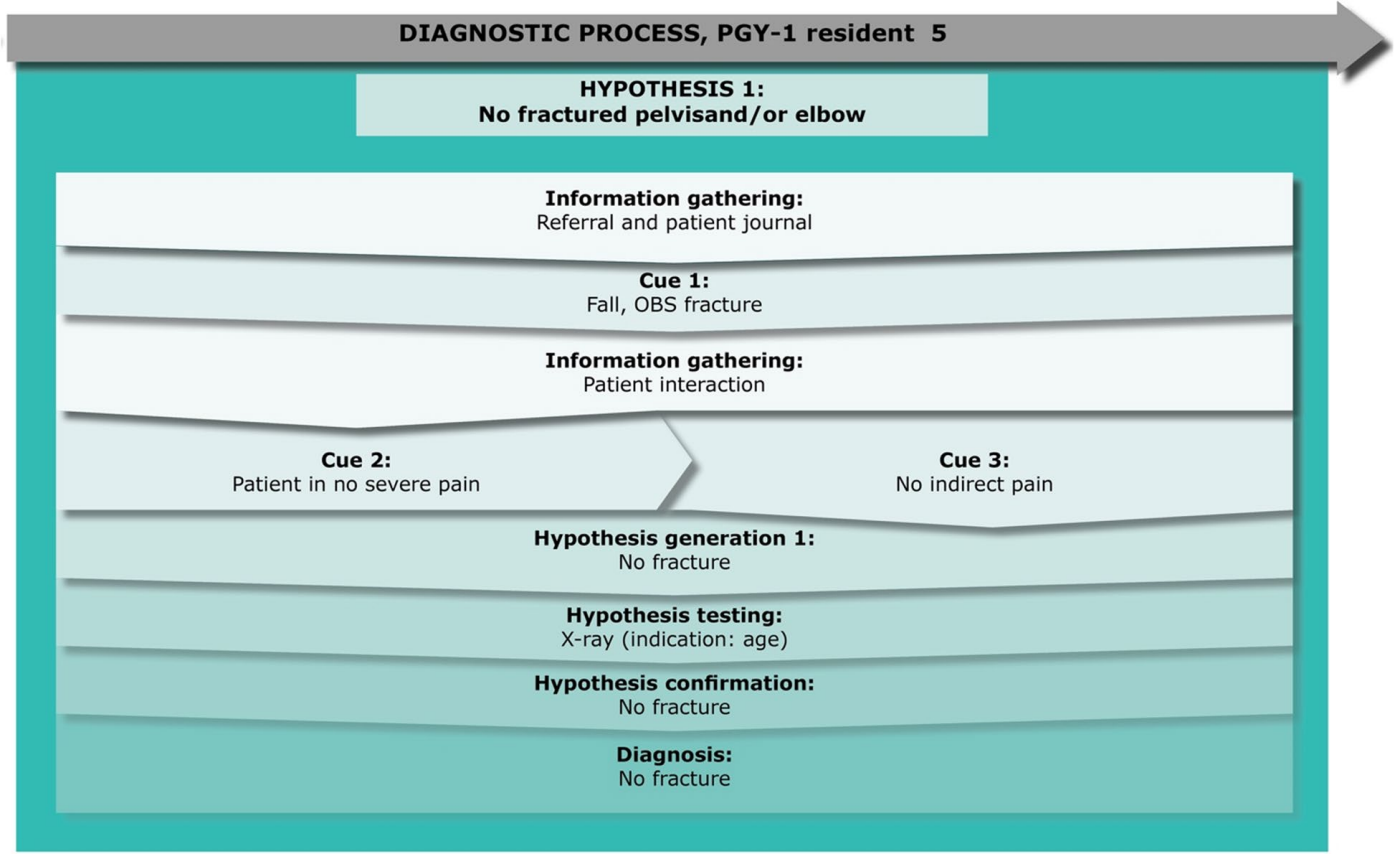
PGY-1 resident 5, “Mark” – a case of fracture

**Fig. 6:**
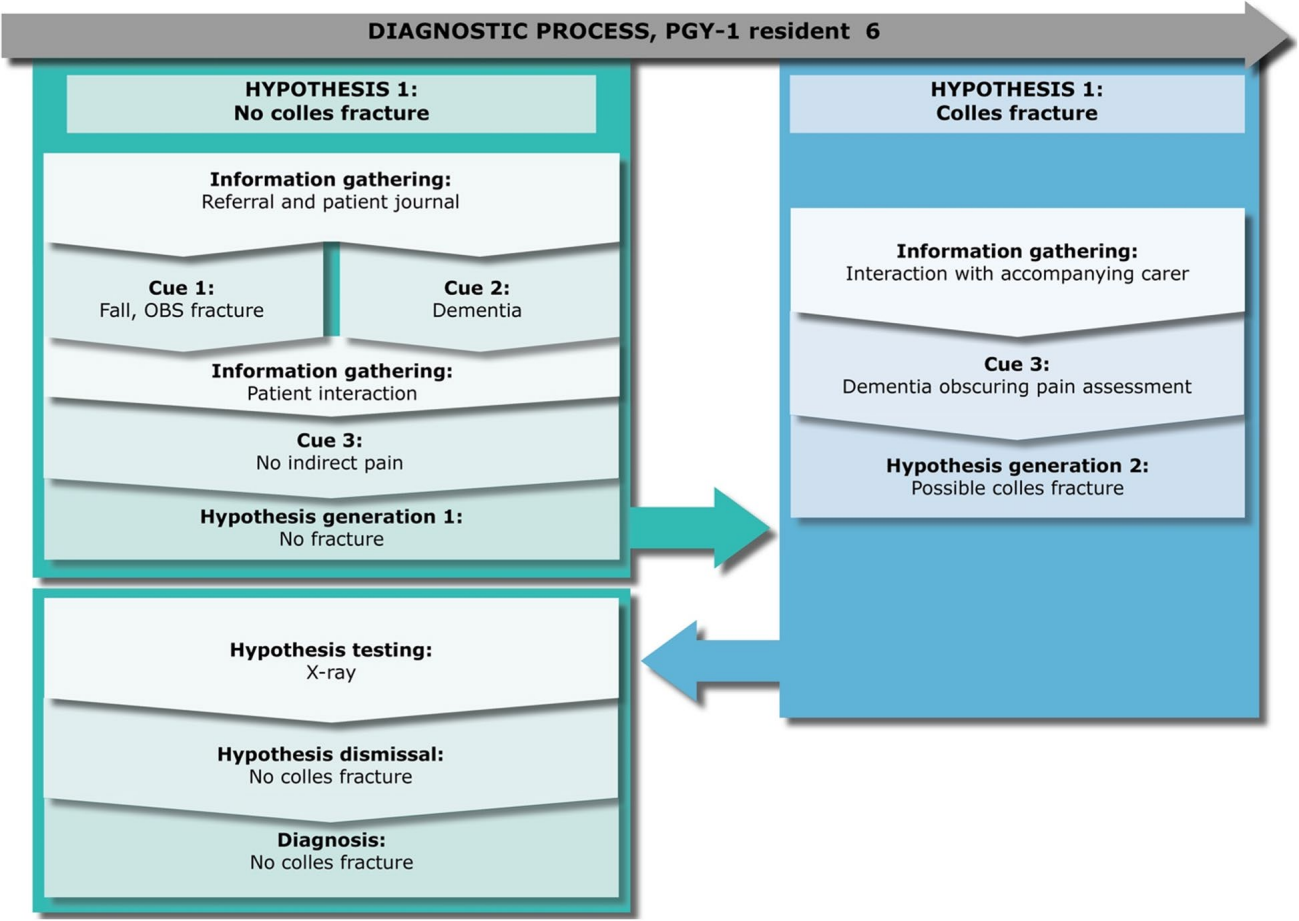
PGY-1 resident 6, “Casper” – a case of fracture

**Fig. 7:**
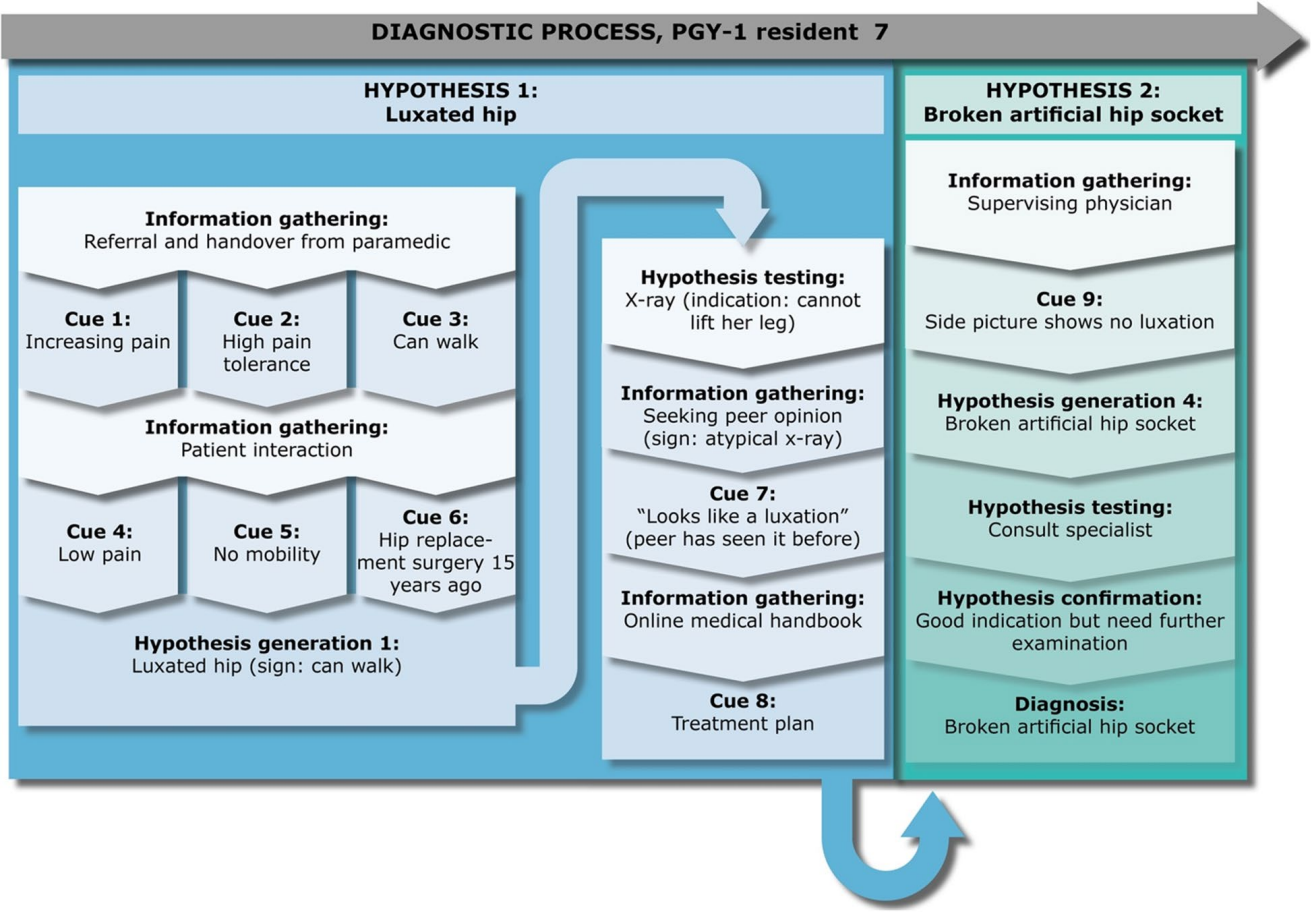
PGY-1 resident 7, “Daniel” – a case of a luxated hip

The narrative analysis of the chronology of the diagnostic process of each informant was visualized into individual models of diagnostic reasoning. These models of diagnostic reasoning are illustrated in Figs. 1, 2, 3, 4, 5, 6 and 7. They show a progression in thought processes and changes in hypotheses (color-coded).

Anne has been in residency for 5 months. In the observed patient encounter, she received a 60-year-old patient who had stepped on a glass a week ago. The patient (Anton) is now referred to the ED with inflammation. In her first hypothesis generation, Anne comments that a physician has not seen Anton before referring him to the ED. This would have been common practice with this type of patient. Thus, her thinking is part of the cue collection in her first hypothesis generation. She reads the short referral note, which states that the patient had stepped on something while swimming in the ocean. The patient’s pain has increased since, and he has a hard time walking. Anne’s initial hypothesis is therefore, that the foot is inflamed. Anne observes that the patient’s walking is affected. However, during her clinical evaluation she rejects her initial hypothesis based on the cues that there is no swelling, redness, or heat, and the observation that he can support on the foot. Therefore, she changes her hypothesis. To check further and evaluate her new hypothesis, she examines the wound more closely, finding no foreign bodies. This way she is confirming that the patient’s pain is due to soreness from a healing wound rather than an inflamed injury. After this diagnostic decision, she goes back to look for the referral note once again. Anne’s behaviour shows signs of a relevant checking behaviour as evidenced by her retrospective comments on her rationale for checking the referral note after seeing the patient:

"*I just want to make sure I have not missed something, since [the referring physician] thought he should come in [to the ED].*"

The case continues with Anne becoming confused as to why the patient was referred to the ED. Anne did not deem that there was a justifiable cause, but she was not confident in acting on this due to her lack of experience.

Ellen has been in residency for almost 3 months. She is receiving a female 83-year-old patient (Mary), who reportedly has fallen in her own home. Initially the referral note suggests indications of a subdural haematoma, which Ellen settles on as her working hypothesis. Ellen thoroughly checks Mary’s history and medication in the electronic patient journal. Based on this investigation, she states that it is a rather classic case and that she is *“fairly sure”* that Mary is suffering from a subdural haematoma, as the home nurse has reported that the patient had high blood pressure and headache. When examining the patient, the emergency medicine technicians (EMT) report that they were only able to detect a small increase in blood pressure, whereas all other critical values are normal. Furthermore, they inform Ellen, that there are no neurological symptoms. This contradicts Ellen’s working hypothesis, and the EMT reports that Mary recently broke her arm, which has influenced her independence and daily routine. In the retrospective interview, Ellen describes how her working hypothesis then changed, thinking that the high blood pressure was due to a stress response. However, during the physical examination, Ellen routinely explores the headache, and it turns out to be rather severe and slowly building. In the retrospective interview, Ellen explains that concussions can be tricky. To be sure, she changes her working hypothesis for the third time, wanting to investigate the probability of a concussion, as she knows from the referral that Mary hit her head when falling. She performs a quick neurological screening and concludes that she is uncertain if the symptoms warrant a CT scan. Therefore, Ellen chooses to seek out a supervising physician, and they discuss that the small increase in blood pressure could be white coat syndrome (fourth hypothesis), but the supervising physician agrees that a CT scan and ECG would be a good idea. Both come out normal and Ellen concludes that Mary’s increased blood pressure is due to white coat syndrome.

Christina has been in residency for 3 months. She is receiving a female 70-year-old patient (Karen) who has twisted her ankle. From reading the electronic patient journal and referral note alone, Christina verbalize that her first hypothesis is that there is a fracture. This is based on the patient history because she has osteoporosis and has previously had fractures related to this disease. However, when Christina meets Karen, Christina becomes uncertain of this diagnosis, as there is only limited swelling and no indirect pain on indicative pressure points. Christina explains in the retrospective interview, that at this point, she only expected a sprained ankle, as none of the symptoms indicated a fracture. However, Christina orders an x-ray despite this hypothesis and no indicative symptoms. She reflects that her rationale builds on the hypothesis that Karen has osteoporosis, indicating a flexible conceptual knowledge, in line with epistemic distance:

*“I usually do that if I have a patient where I’m like: should or should I not? Then I’m like: okay would I be able to go to sleep tonight without thinking about it?”*

This is thus a strategy of using her emotional response to scaffold self-regulation when she experiences gaps in knowledge (epistemic distance). Consequently, from this x-ray she notices a possible fracture and changes her diagnosis back to the original and she seeks out a specialized physician to get her hypothesis confirmed.

It is an evening shift and Julie, who is in her third month of residency, is taking over a male, 73-year-old patient (Karl) from the other resident, Daniel, who is looking at his notepad and reciting information. Daniel explains that Karl had fallen during clean up from a celebration dinner and cut himself on his lower leg. Daniel explains that he has already conferred with a supervising physician, who said to sew the open wounds and admit Karl, as he had many comorbidities. Julie agrees with the Daniel that Karl should be admitted due to his injuries. Then Daniel interjects that Karl is also suspected to be intoxicated. Daniel explains that he is tired and apologizes for providing unorganized information. By the end of the handover a supervising physician, who has a surgical specialty, is interrupting the handover, arguing that they should discharge Karl. As this supervising physician continues to interrupt the handover, Julie retrospectively describes her experience with these interruptions:

*“Then the supervising physician comes in and says that if there has been given a plan [by a supervisor], one should never continue to ‘shop around’… but I feel like… if there have been over 12 new patients since then, then I want to make a new evaluation, and then I will have to consult again.”*

She adds to this, describing the emotional impact on her performance:

*“So, I feel like giving up. [Like] am I alone, then? Just because [the previous resident] has already conferred the patient with another supervising physician? In addition, what if I make a new judgement that is not consistent with that… I feel uncomfortable backing a decision I did not make… [so I] become irritated because it is arrogant [of this supervising physician to put me in that position] … the feeling of being a little helpless…. powerless is a good word for it.”*

After this chaotic handover, Julie decides to go see Karl and from this interaction, she verbalizes in the retrospective interview that she immediately recognises that Karl is lucid, and has no severe pain. Therefore, she hypothesizes that there is no need for admitting him as the injuries are only superficial. She seeks out another supervising physician to have him see Karl and assess the severity of his injuries. This supervising physician agrees with her diagnosis and plan for treatment (sewing and dressing the wound) and her decision to discharge Karl.

Mark is in his 5th month of residency and is treating a 75-year-old female patient (Eden). He does not have time to read the patient journal on Eden, as he priorities getting the handover from the EMTs. Mark knows from the referral note that the patient has fallen and remarks while walking to the patient room that he should always expect a fracture in the hip or pelvis area, due to the age and injury. However, during the patient interaction, he quickly recognizes that there is no indicative pain, and his first hypothesis is that there is no fracture to her pelvis or elbow area (which were the areas mentioned by the EMTs). Mark decides on performing an x-ray to be sure, due to Eden’s age, which confirms his hypothesis and he diagnoses her with no fractures to the pelvis or elbow and discharges her.

Casper is in his third month of residency and is seeing a 72-year-old male patient (Hans) diagnosed with dementia. Hans has fallen and Casper suspects he has a Colles’ fracture. Furthermore, Hans’s leg is rotated, which Casper mentions, supports the diagnosis of Colles’ fracture as it is a sign of such a fracture. While Casper is reading up on Hans’ case, a nurse interrupts several times, which Casper comments on retrospectively:

*“You become removed from your line of thoughts… the process that you are in.”*

As a result, Casper does not settle on a hypothesis before seeing Hans. While reading the electronic patient journal, Casper is thorough, and despite noting several relevant pieces of information that could indicate a fracture, he verbalizes that he should ‘investigate’ or ‘be suspicious of’ several different symptoms. However, Casper still does not settle on a hypothesis and several times iterates that *“we will know when we see the patient”* or *“then I know I will need to be extra thorough”* indicating that he uses this preparation more as an overview and way of prioritizing his investigation, rather than narrowing down the hypothesis. Casper goes to examine Hans who is accompanied by a caregiver from his residential facility. In the retrospective interview, Casper comments that he initially suspects that there is no fracture based on the physical examination, but that this hypothesis is disrupted by a conflict with the caregiver. Casper informs the caregiver that he does not suspect a fracture and will possibly discharge Hans. The caregiver protests and Casper comments retrospectively:

*“She might be worried. Because she has previously experienced that the ED overlooked a fracture on one of the other senior residents whom she cared for… Nevertheless, I will not order an x-ray. Although she is very worried… If I examine the patient and cannot find anything, [then I will not order an x-ray]. Otherwise, I could just order an x-ray without seeing the patient.”*

Here, Casper is mindful of a conflict of interest. Nevertheless, he is also aware of his role as the physician and the authority associated with it, using this as justification for his choice to maintain his autonomous professional judgement:

*“I think it is my responsibility [to take the lead]. I’m the doctor so I’m the one in charge.”*

However, as the caregiver continues to question his decision, Casper gives in to this pressure and orders an x-ray. This x-ray confirms Casper’s initial hypothesis and his diagnosis of no Colles’ fracture, and therefore discharges Hans.

Daniel is in his fourth month of residency and is receiving an 83-year-old female patient (Lisa). The referral note suggests a luxated hip. Daniel looks at the coordinating screen and notices that she has already arrived. He says to MLG “*I will look at the patient journal afterward. I would like to get a handover from the EMTs”,* and rushes to the patient room. When he arrives, he greets Lisa and turn his attention to the EMTs. The EMT explains that Lisa is experiencing increasing pain and while she can walk, they assess that she has a high pain tolerance. When examining the patient, Daniel identifies low mobility. Despite Lisa describing low pain, his first hypothesis is that she has a luxated hip, as Lisa also informs him that she had hip replacement surgery fifteen years ago. As seen with Mark, Daniel also reflected upon his role and was cognizant of his inexperienced disposition in this regard. Lisa asks Daniel if it could be due to her hip replacement, and he responds:

*“I have to be honest and say that I don’t know [about the risks of 15-year-old hip replacement surgeries,]”*

Adding in retrospect that:

*“I shall in no way pretend I know more than I do. It neither serves me, nor the patient.”*

Daniel chooses to order an x-ray to confirm his hypothesis. However, when the x-ray presents an atypical picture, he seeks out a second opinion from another residents, Ellen, who happens to be in the office. Ellen explains that she has seen this kind of injury before. To her, it looks like a luxation. However, Daniel is still unsure about the proper diagnosis and choses to consult an online medical handbook on typical treatment plans for a luxated hip. Finally, he settles on the hypothesis of a luxated hip. This happens despite him retrospectively reflecting the following:

*“[B]ecause we fail to look at the x-ray picture of the side profile, where we would expect that the hip joint was outside of the socket, we mistakenly think that it is a luxated hip.”*

Reflecting on his consultation with Ellen, he explains:

*“[I have] blind faith in her, because she is very convincing and has seen patients with luxated hips before… I haven’t seen it before… she has seen it before. She simply looked at the picture and said, ‘it clearly looks like it’s luxated’. So I just jump on that conclusion.”*

Daniel continues to plan the treatment and routinely goes to confer his decision with the supervising physician to get confirmation of his diagnosis and plan. Now, Daniel shows the x-ray pictures to the supervising physician, who disagrees with Daniels’s diagnosis. He asks to see the side profile and identifies that the plastic liner of the artificial hip socket has broken. Daniel explains in the retrospective interview: “*I have never heard of a plastic liner before he mentioned it*”. The supervising physician calls to consult an orthopaedic specialist and confirms the hypothesis and they settle on this diagnosis. Subsequently, Lisa is transferred to the orthopaedic department for further examination.

### Adaptive expertise and diagnostic reasoning

**Fig. 8:**
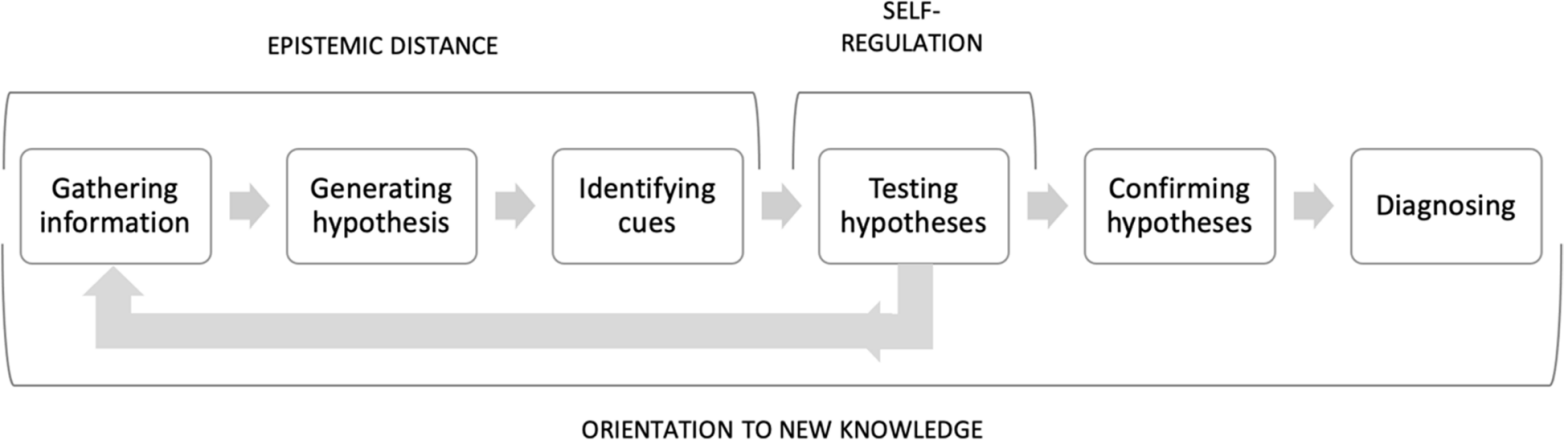
Steps of the adaptive expert diagnostic process

Setting this organization of the residents’ diagnostic process in perspective of the adaptive expert framework, we were able to identify how adaptive practices were temporally distributed in the diagnostic process as illustrated in Fig. 8:

Here, epistemic distance would often occur during processes of information gathering, generating hypotheses, and identifying cues, which were present in the first part of diagnostic processes. Epistemic distancing would help residents become aware of gaps in knowledge and assess when they possessed enough information to move on. Thus, it was observed overlapping cognitive processes identified in the protocol analysis of critical thinking, reflection, and bias reduction. Self-regulation was then seen primarily during the hypothesis-testing process. Here, residents as adaptive experts were able to identify this discrepancy between their existing knowledge and the problem at hand, redirecting their attention back to gathering more information. This concept overlapped cognitive processes of choosing and prioritizing, identified in the protocol analysis. Lastly, orientation to new knowledge was observed as a general attitude or continuing process amongst some residents. It often overlapped with cognitive processes of seeking, explaining or concluding on information which occurred at all times during the diagnostic process. As such, it indicates a continual process of reasoning during diagnostic processes. This reasoning then acts as an indicator of when they cannot explain the information at hand, which may incite curiosity. Importantly, curiosity was not identified as a cognitive concept in the protocol analysis, but behaviours resembling this were observed in during the sensitizing analysis of adaptive expertise. For example, this was observed in Daniel’s case, where he made many efforts to seek out information, as he could not sufficiently explain the symptoms from the information he had. This curiosity led him to consult peers, supervisors, and medical handbooks, describing how he wanted to know more. Ultimately, he explains that the patient’s diagnosis was new to him. It came as a surprise to him, and he spent a long time with the supervisor to look over the x-rays to understand the condition better.

## Discussion

This study sought to explore how adaptive expertise permeated diagnostic processes within medical residents in encounters with geriatric emergency patients. Results showed that all residents displayed adaptive cognitive practices during the diagnostic process. This finding provides arguments for novices being capable of adaptive expert cognition, thus extending the understanding of the development of expertise [37]. Adaptive expertise has been conceived as a learnable output of training, using the rhetoric ‘becoming an expert’ [38]. By integrating the adaptive expert framework in diagnostic decisional processes, and looking at how adaptive expert cognition was temporally distributed throughout, this study showed that novices sometimes were able to utilize adaptive expert cognition similarly to experts [37], but that they did not do so consistently. This suggests that adaptive expertise may be an available potential, but not yet a consistent and reliable competency within residents. Results also showed that some residents were more prone to being affected by their context, like peers’ suggestions and opinions, or uncertainty, as when they were questioned on their authority. This finding suggests that the development of adaptive expertise is also fostered by individual predispositions and is in line with previous research [25, 70, 71] showing that attitude and professional identity inherently affects the development of adaptive expertise. The study demonstrate how these factors are especially important for residents.

### Key point 1: Epistemic distance and self-regulatory processes are a part of hypothesis generation, and hinder premature closure

As seen from Figs. 2, 4, 5, 6 and 7, some residents identified fewer cues before the patient meeting, which made them liable to change their hypothesis several times before collecting adequate information to confirm their hypothesis. Some residents (e.g., Ellen or Julie) prematurely tested their hypothesis, and they were forced to repeat the whole cycle when proven wrong. On the other hand, when residents collected more cues in their initial information-gathering process (e.g., Daniel) or when the patient case was relatively simple (e.g., Mark), fewer shifts in hypothesis were necessary before confirming their diagnostic hypothesis. Arguably, when comparing these two methods of arriving at a diagnosis, premature closure on informational cues led to a more laborious diagnostic process for these informants.

Another possible reason might be that residents are primed by reading the patient journal. As we saw, some residents prematurely anchored to one specific diagnosis and need thorough discouragement (e.g., x-ray) in order to put this hypothesis away, despite seeing the patient.

In the case with Mark, he does not have the time to read the patient’s journal before examining her, and quickly arrives at the right hypothesis from the examination and handover from the EMTs. In this case, Mark is scaffolded by his environment to be more open to information. Strategies for avoiding premature closure were seen in Casper’s case. Thus, Casper explicitly states that he is gathering an overview, but is withholding a fixed hypothesis before seeing the patient.

These differences detected in cue collection impacted hypothesis generation. Residents who were less epistemically aware in their initial hypothesis generation, were prone to premature closure on a hypothesis, making them more prone to needing to change hypothesis. This rapid hypothesis generation has been shown to entail a risk of committing errors [11]. However, while Elstein and Schwarz [44] argue that novices struggle with generating hypothesis and planning because they have difficulty moving beyond data collection. This study indicates that the residents were able to move beyond data collection, and in some cases move towards rapidly changing hypotheses. Results showed that the challenge resided in the quality of hypotheses, as also argued by Elstein and Schwarz [44]. This could suggest that the observed residents had enough experience to move beyond data collection and enough knowledge to form a hypothesis. However, the sometimes-low quality of the hypothesis could indicate that at this point in their professional development, they have not been trained to critically reflect on their experiential acquisition of knowledge.

Results indicated a lower tolerance for information load amongst residents, as illustrated by Casper, who wards off a nurse when preparing for his patient, saying: *“I cannot handle more patients right now”,* explaining retrospectively *“it’s difficult to have it all in your head”*. Critical thinking skills and metacognition has been argued to improve cognitive efficiency through the reconstruction of knowledge, which is needed to free mental capacity or position to perform adaptive expertise [72]. Here, context specificity helps increase the residents’ tolerance of information load [72]. This might help residents free mental capacity to ‘slow down’ and improve their diagnostic reasoning [45]. Thus, such strategies might be imperative to employ in early residency, so as to not become overloaded, which would impede decisional competency.

While we have at this point argued for the personal nature of these cognitive competencies, research within the framework of adaptive expertise, clearly points to a collaborative nature of CDM [35, 72]. That a cognitive competence stems from a collaborative effort, but is enacted by the individual physician should not be a contradiction. However, there might be a lack of operationalisation of the notion of self-regulation. This study might add insight into the nature of self-regulation in adaptive expertise is. We identified prioritization and choosing as cognitive features occurring during self-regulation (re-directing), which bear some resemblance to the concept of self-regulated learning [73], with a focus on planning, goal-setting, and visualisation. Thus, this study suggests that future studies could benefit from further investigating the interconnections between these two concepts.

### Key point 2: Emotional responses and uncertainty influence resident performance

The results indicate that residents under some conditions can apply epistemic distance, but was seemingly affected by their confidence in their hypothesis. Christina only acted on her epistemic distance, due to her expected negative emotional response, whereas Daniel expressed his lack of knowledge of the patient’s problem and made several attempts at self-regulatory behaviour in seeking out information and peer feedback. This could also be an indication of uncertainty present amongst many of the residents. When residents were uncertain and timid about their knowledge and competencies, they required additional reassurance of their assessment before taking action. The actions they took included weighing risks, conferring guidelines or inquiring about the patients’ need or peers’ opinions. These can be seen as epistemic behaviours. However, checking behaviours were also recorded and they seemed often to be initiated by an emotional response such as fear of missing a diagnosis, as seen in Anne’s case.

Disturbances could add to this uncertainty. The case of how Casper was affected by pressure from his context illustrates how residents take on their role through their actions in the clinical setting, and what the clinical setting allows. As described in his case, he retrospectively justifies and solidifies his authority by the role that he has as a physician in the department, but when pressed by the caregiver, gives in and becomes unsure of his initial hypothesis.

As described in the results of the protocol analysis some residents viewed their role as a physician as tied to strategies used for being epistemically aware. Instances such as described in Julie’s emotional response, influenced how residents experienced the learning culture in the department. Consequently, Julie did not feel comfortable with the available support from the first supervising physician and sought out another physician to consult for small corrections to the diagnostic process throughout. This demonstrate the reciprocity of cognition and emotion [74] and underscores the importance of considering emotional regulation and identity in professional development. Residents who verbalized their role as physicians would pick up on cues in their context that were not directly related to their diagnostic reasoning. This would be seen in instances involving conflicts of interest or disturbances. As a result, they would continuously check the appropriateness of their diagnostic reasoning and become more uncertain. Research has shown that uncertainty tolerance does reduce errors [75–77] and being able to tolerate unpredictable circumstances in an adaptive expert manner, helps residents engage in learning opportunities [70, 78]. Research also suggests that the main concern for educational interventions should concern rational failure, which is caused by individual affective and cognitive biases. Workplace factors increasing cognitive load and limited resources further increase the risk of such biases [55, 79]. Researchers argue that critical thinking and metacognition reduce affective biases [80, 81], and reduce errors in diagnostic processes [82]. Our data showed that critical thinking and self-regulation were processes that residents actively engaged in. However, conceptually, these are not commonplace competencies, and researchers in medical education argue that these must be cultivated through education [70, 78].

While all residents in this study utilized critical thinking, only one resident reflected on her emotional response and how emotions influenced her decision competency. This suggests that affective biases are not recognized in this particular setting, in that verbalization of emotional responses is not cultivated. Therefore, how the physician-environment fit takes part in cultivating critical thinking and reflective practices which encompass emotional features and how they affect residents, could help reduce the mortal consequences of biases in diagnostic reasoning.

Being epistemically aware would help residents be more thorough in the initial parts of the diagnostic process, leading them to be open to new information when collecting cues and gathering information. As discussed in the beginning, efficient pattern recognition comes with experience [1–3, 44], however, novices do not yet possess enough experience to do that. Thus, they are reliant on hypo-deductive reasoning, as evident from the results. However, as we saw when applying the adaptive expert framework, being epistemically aware help gather information and be open towards new information, which may prevent premature closure based on inadequate information, and thus lead to less changes in working hypothesis. We saw that when residents moved on from information gathering too fast, they could be forced to make assumptions based on too little information, leading them to change hypothesis. The adaptive expert framework highlight openness [25, 45] and the ability to be comfortable with not knowing [83]. Reaching for a hypothesis and testing it too fast, could be a sign of an inability to be at peace with, and lean into, uncertainty. Supporting self-regulatory processes is key when then testing hypothesis, as realising that being wrong can impact their ability to redirect themselves to learn more about the problem at hand. In order to not let such experiences impede professional development, senior staff must model allowing and handling such emotions [84]. It is therefore imperative to foster a culture that sees professionalism as encompassing being aware that they do not know everything, that it is okay to change their mind, and that being curious is important, and these are foundational cognitive skills for medical professionals.

### Key point 3: The way residents assume their role in the ED affects their cognitive strategies

Results showed that while all residents reflected on their professional role, they were often verbalized through cultural expectations of their role as a physician in general, and not specific to their emergency context. Their decisional competency was extended through this negotiated general authority. This suggests the need for investigating settings that allow residents to acknowledge knowledge gaps, without feeling that their professional identity and authority are being challenged. Uncertainty can be interpreted as a sign of incompetence [85], and research shows that trainees often feel the need to stage a performance [86]. This study suggests that supporting residents to develop a professional identity which allows knowledge gaps could scaffold adaptive expert cognition. This is in line with studies investigating the link between uncertainty and decision-making, arguing that recognizing and classifying uncertainty can help us consider new perspectives and acquire relevant knowledge [87]. At the same time, results indicate that junior physicians needed to clarify their professional roles and expected competencies could facilitate such dialogue in line with current research [33, 86]. Thus, professional development is supported when senior personnel clearly tell residents what is expected of them [86] and incorporate variations into learning situations to train residents’ ability to adapt and handle complexity and ambiguity [33].

Our results showed that residents tended to rely on contextual cues to prompt their adaptive expert cognition. Previous research has shown that this strategy can increase cognitive load [55, 72]. Results suggested that some residents required contextual structures like structured examinations to scaffold their ability to employ adaptive expert cognition, which has not previously been described in the literature on adaptive expertise.

In summary, residents used a range of information and cognitive processes during diagnostic decision-making. All informants demonstrated adaptive expert cognition, but the chronological approach to analysis, showed how these were interspersed with routine practices and that disturbances and attitude impacted their diagnostic reasoning process. Results demonstrated that adaptive expert cognition was exerted at different times throughout the diagnostic process, indicating that the cognitive features of the adaptive experts´ framework are embedded in the decisional process, differently.

### Strengths and limitations

Introspection is a well-researched methodology [56] and we acknowledge that observing first-order thoughts through verbalization is difficult [88]. The method of Think-Aloud interviews was chosen in order to access second- and third-order verbalizations of thoughts [47]. As such, the choice of including both concurrent and retrospective interviews served to obtain data as close to thought processes as possible [49, 50].

#### Changes and differences in concurrent and retrospective interviews

The choice of using both concurrent and retrospective think-aloud interviews had both strengths and some limitations. The use of both served to check for the reliability of the findings, by having time to transcribe and make initial analytical interpretations before the retrospective interview, which could then be confirmed or denied by the informant. Furthermore, the retrospective interview provided more thorough and unaffected insights into the informants’ reasoning, while allowing for minimal invasion during the concurrent interview. This was especially worthwhile in the natural setting, as the concurrent interview did not interfere with flow management too much. However, during the retrospective interviews, some informants would express that “I should have done this…” or “it is probably because I thought…”, indicating, firstly, that the retrospective reasoning might not reflect the actual thinking processes during the patient encounter, but rather their procedural knowledge. This is in line with prior studies investigating the use of both concurrent and retrospective think-aloud interviews, arguing that retrospective interviews are prone to errors due to recall biases and memory decay. This study adds to these findings, arguing that retrospective recall was biased but counteract Whitehead et al.’s [89] finding that concurrent think-aloud provides more rich data, in that we found valuable insights from the retrospective think-aloud. One key difference was that informants in our study were specifically instructed not to provide rationales during the concurrent interview. Secondly, a bias in the data may arise from the fact that we had no means of assuring that the reasoning that the informants provided during the retrospective interview, reflected their actual reasoning in the natural setting, and was not just a consequence of prompting retrospective verbalization of their thoughts. This point has previously been discussed in the think-aloud procedure and was the reason for including both concurrent and retrospective think-aloud interviews. However, this study found that this limitation was still present, despite performing both, and as the method of including both is immensely time-consuming and demanding of the researcher, it is reasonable to discuss the cost–benefit of using this methodology in opposition to common think-aloud methods or even interview methods. To this point, we found that performing concurrent think-aloud interviews was feasible and when asked in the retrospective interview, the informants in this study did not find it constraining, nor did they add new types of information not already addressed during the concurrent think-aloud interview.

### Conclusion and implications

This think-aloud interview study showed that resident CDM and adaptive expert cognition are closely related. They are both affected by uncertainty and professional role, in how residents assumed confidence in their authority by either the cultural expectation or their own merits as an ED physician.

The concurrent and retrospective data established that residents who were less able to detect their knowledge gaps, too quickly settled on a hypothesis. This not only meant that they were forced to revise the initial hypothesis during the patient encounter, but it reflected a tendency to forego adaptive expert practices, in change for decisional action. Research on adaptive expertise needs to be able to explain how residents’ thoroughness in hypothesis generation is a consequence of their confidence and tolerance of restrictions in knowledge and uncertainty. Results showed that residents could apply adaptive expert cognition during diagnostic reasoning, and that epistemic distance was aligned with the information-gathering process, whereas self-regulation applied to the hypothesis-testing process. Furthermore, results underscored the importance of viewing the professional role, attitude, and beliefs as intermixed during the entire diagnostic process, which might lead to physicians’ openness and orientation towards new learning opportunities.

### Future directions for research

This study adds to the research on residents’ cognitive decision-making processes. However, given the relationship between residents’ diagnostic reasoning and their adaptive expert cognition, future research might focus on applying the adaptive expert framework to training diagnostic reasoning. Specifically, how do we train to reduce uncertainty, by means that are not mere knowledge acquisition? Experience is not sufficient to rid novice residents of uncertainty and give them the tools to act adaptively. Therefore, future studies could operationalize findings in this study of the possible positive effects of learning to accept knowledge gaps as a natural part of medical practice, and methods of slowing down in the initial hypothesis generation.

## Supplementary Information

**Additional file 1.**

## Data Availability

The raw datasets analysed during the current study are not publicly available due to General Data Protection Regulation in respect to the informants and patients but anonymized raw data are available from the corresponding author on reasonable request. Anonymized accumulated data are included in this published article.

## Abbreviations

ED: Emergency Department
PGY-1: Postgraduate year 1
CDM: Clinical Decision-Making
EMT: Emergency Medical Technician
