# Supplementary material for: Clinical decision-making and adaptive expertise in residency: a think-aloud study
Source: BMC Med Educ. 2023 Jan 12;23:22. doi: 10.1186/s12909-022-03990-8 (PMC9835279; doi:10.1186/s12909-022-03990-8)
Supplement: Supplementary file 1 — Additional file 1. [file 12909_2022_3990_MOESM1_ESM.docx]

Additional file 1: Informational concepts and cognitive process codes

# Table 1: Informational concept codes

| **#** | **Concepts** | **R1** | **R2** | **R3** | **R4** | **R5** | **R6** | **R7** |
| --- | --- | --- | --- | --- | --- | --- | --- | --- |
| 1 | Actions | 1 | 1 | 1 | 1 | 1 | 1 | 1 |
| 2 | acuteness |  | 1 |  |  | 1 |  |  |
| 3 | Admission |  | 1 |  | 1 |  |  | 1 |
| 4 | age-related symptoms |  | 1 | 1 | 1 | 1 | 1 | 1 |
| 5 | Algorithms/guidelines/tools |  | 1 | 1 | 1 | 1 | 1 | 1 |
| 6 | Approach/agenda |  | 1 | 1 | 1 | 1 | 1 | 1 |
| 7 | Behaviour (own) |  |  | 1 |  |  |  |  |
| 8 | Causality | 1 | 1 | 1 |  | 1 |  |  |
| 9 | comorbidity |  |  | 1 | 1 |  | 1 |  |
| 10 | Complexity |  |  | 1 | 1 |  | 1 |  |
| 11 | computer | 1 | 1 |  |  |  | 1 | 1 |
| 12 | Concurrent affiliations with other medical teams |  | 1 |  |  | 1 |  |  |
| 13 | Conflict with patient/next of kin/peer expectations/perspective |  |  |  | 1 | 1 | 1 |  |
| 14 | contradicting information | 1 | 1 | 1 | 1 | 1 |  | 1 |
| 15 | consent (patient/next of kin) |  |  |  |  |  | 1 |  |
| 16 | culture (verbalizing/creating) |  |  |  | 1 |  |  |  |
| 17 | department specific knowledge/structure | 1 | 1 | 1 | 1 |  |  |  |
| 18 | diagnosis | 1 | 1 | 1 |  | 1 | 1 |  |
| 19 | diagnostic codes |  |  | 1 |  |  |  |  |
| 20 | differential diagnosis |  | 1 | 1 |  |  |  | 1 |
| 21 | discharge from hospital |  | 1 |  | 1 |  | 1 |  |
| 22 | Distractions (presence or handling of) |  |  | 1 | 1 |  | 1 |  |
| 23 | elderly (age, frailty, mobility) |  |  | 1 | 1 |  | 1 | 1 |
| 24 | Emergency medicine |  | 1 |  | 1 |  |  |  |
| 25 | Emotions |  |  |  | 1 |  |  |  |
| 26 | Novice role |  |  | 1 | 1 | 1 | 1 | 1 |
| 27 | experience (impact on performance) |  | 1 | 1 | 1 | 1 | 1 | 1 |
| 28 | general/common knowledge | 1 | 1 |  | 1 |  | 1 | 1 |
| 29 | habit | 1 |  | 1 | 1 | 1 |  | 1 |
| 30 | Handling/experiencing uncertainty (own) |  |  | 1 | 1 | 1 | 1 | 1 |
| 31 | handover |  | 1 |  | 1 | 1 |  | 1 |
| 32 | Humour |  |  |  | 1 |  |  | 1 |
| 33 | hypothesis | 1 | 1 | 1 |  |  | 1 | 1 |
| 34 | information lacks | 1 | 1 |  |  |  |  | 1 |
| 35 | Interprofessional collaboration/coordination |  | 1 | 1 |  | 1 | 1 | 1 |
| 36 | Interprofessional opinions | 1 | 1 |  |  | 1 | 1 |  |
| 37 | information sources |  | 1 | 1 | 1 | 1 | 1 |  |
| 38 | intuition/gut feeling/clinical gaze |  |  | 1 | 1 |  | 1 | 1 |
| 39 | knowledge (academic/theoretical) |  | 1 | 1 |  | 1 | 1 | 1 |
| 40 | Medical art/role expectations |  |  |  |  |  | 1 |  |
| 41 | Medicine |  | 1 |  | 1 | 1 | 1 | 1 |
| 42 | methods of obtaining more knowledge/information |  | 1 | 1 | 1 | 1 | 1 | 1 |
| 43 | Needs (own) |  |  | 1 |  |  | 1 |  |
| 44 | new symptoms/information |  | 1 | 1 | 1 | 1 |  | 1 |
| 45 | Normal practice |  |  | 1 | 1 | 1 |  | 1 |
| 46 | Organizational knowledge/structure | 1 | 1 | 1 |  |  | 1 |  |
| 47 | Overview/summary | 1 | 1 | 1 | 1 | 1 |  |  |
| 48 | Pain |  | 1 | 1 | 1 | 1 | 1 | 1 |
| 49 | patient characteristics/pathology | 1 | 1 | 1 | 1 | 1 | 1 | 1 |
| 50 | Patient experience/perspective/knowledge | 1 | 1 | 1 | 1 | 1 | 1 | 1 |
| 51 | Anamneses (patient history) | 1 | 1 | 1 |  | 1 | 1 | 1 |
| 52 | patient journal | 1 | 1 | 1 | 1 | 1 | 1 | 1 |
| 53 | patient’s social circumstances | 1 | 1 | 1 | 1 | 1 | 1 |  |
| 54 | peer opinions |  | 1 |  | 1 |  | 1 |  |
| 55 | Plan | 1 | 1 | 1 | 1 | 1 | 1 | 1 |
| 56 | practice of other healthcare professionals/departments | 1 | 1 | 1 | 1 | 1 | 1 | 1 |
| 57 | preference (own) |  | 1 |  | 1 | 1 | 1 | 1 |
| 58 | prior medical evaluations |  | 1 | 1 | 1 |  | 1 | 1 |
| 59 | Principals/ideals |  | 1 | 1 | 1 | 1 |  | 1 |
| 60 | professionalism |  |  | 1 | 1 | 1 | 1 | 1 |
| 61 | Quality of information |  |  | 1 |  |  | 1 | 1 |
| 62 | Referral |  | 1 | 1 | 1 |  | 1 |  |
| 63 | requirements for diagnosis | 1 | 1 | 1 | 1 | 1 | 1 | 1 |
| 64 | Responsibilities |  | 1 | 1 |  | 1 | 1 | 1 |
| 65 | risks and misdiagnosis |  |  |  | 1 | 1 | 1 | 1 |
| 66 | Role as an emergency physician (own/others') |  | 1 | 1 | 1 | 1 | 1 | 1 |
| 67 | safety net/safety measures |  |  |  | 1 |  |  |  |
| 68 | scientific evidence | 1 | 1 | 1 |  | 1 |  |  |
| 69 | severity |  | 1 |  |  |  |  |  |
| 70 | sign/patient state |  | 1 | 1 | 1 | 1 | 1 | 1 |
| 71 | tests | 1 | 1 | 1 | 1 | 1 | 1 | 1 |
| 72 | their own biases |  | 1 | 1 |  | 1 | 1 | 1 |
| 73 | time |  | 1 | 1 | 1 |  | 1 | 1 |
| 74 | treatment |  | 1 | 1 | 1 |  | 1 | 1 |
| 75 | Triage | 1 | 1 | 1 | 1 | 1 |  | 1 |
| 76 | value |  |  | 1 | 1 | 1 |  |  |
| 77 | Wording | 1 | 1 | 1 | 1 | 1 | 1 | 1 |
| 78 | Workflow/workload/efficiency |  |  | 1 |  |  |  |  |
| **Occurrences in all** | | **24** | **53** | **56** | **54** | **46** | **51** | **47** |

# Table 2: Cognitive process codes

| **#** | **Operator** | **R1** | **R2** | **R3** | **R4** | **R5** | **R6** | **R6** |
| --- | --- | --- | --- | --- | --- | --- | --- | --- |
| 1 | Alignment of expectations (patient/peers/healthcare professionals) |  | 1 | 1 | 1 |  | 1 |  |
| 2 | Assess acuteness |  | 1 |  |  | 1 |  |  |
| 3 | Assessing role alignment |  |  |  |  | 1 | 1 | 1 |
| 4 | Assessing responsibility |  |  |  | 1 | 1 |  | 1 |
| 5 | Assessing risks/severity |  | 1 |  |  | 1 | 1 | 1 |
| 6 | automated/routine behaviour |  | 1 | 1 |  | 1 | 1 | 1 |
| 7 | biases |  |  | 1 | 1 |  | 1 | 1 |
| 8 | Bias-reduction strategies |  | 1 | 1 |  |  |  |  |
| 9 | checking | 1 | 1 | 1 | 1 | 1 | 1 | 1 |
| 10 | Chose | 1 | 1 | 1 | 1 | 1 | 1 | 1 |
| 11 | communication (verbal/nonverbal, peers/patient) |  | 1 | 1 | 1 | 1 |  | 1 |
| 12 | conclude | 1 | 1 | 1 | 1 | 1 | 1 | 1 |
| 13 | cue identification/action | 1 | 1 | 1 |  |  | 1 | 1 |
| 14 | Culture (alliance/contribution in creating a culture) |  |  |  | 1 |  |  |  |
| 15 | Critical thinking/assessing quality of information or source of information | 1 | 1 | 1 | 1 | 1 | 1 | 1 |
| 16 | deciphering point of departure |  |  | 1 | 1 | 1 | 1 | 1 |
| 17 | deciphering the relevance of findings to mediate the diagnostic process |  | 1 | 1 | 1 |  | 1 | 1 |
| 18 | Deciphering relevance/fit to department | 1 | 1 |  |  | 1 |  |  |
| 19 | epistemic distance/gap in knowledge |  | 1 | 1 | 1 | 1 |  | 1 |
| 20 | explain | 1 | 1 | 1 |  | 1 | 1 | 1 |
| 21 | Gathering thoughts/summarizing/gaining overview |  |  |  | 1 |  |  |  |
| 22 | heuristic |  | 1 | 1 | 1 | 1 | 1 | 1 |
| 23 | hypothesis confirmation/dismissal |  | 1 | 1 |  | 1 | 1 | 1 |
| 24 | hypothesis generation | 1 | 1 | 1 |  | 1 | 1 | 1 |
| 25 | hypothesis testing | 1 | 1 | 1 |  | 1 | 1 | 1 |
| 26 | identifying needs (own/patient) | 1 |  | 1 | 1 | 1 | 1 | 1 |
| 27 | information seeking/collecting information from prior examinations | 1 | 1 | 1 | 1 | 1 | 1 | 1 |
| 28 | Information sorting |  | 1 | 1 | 1 | 1 |  | 1 |
| 29 | (inter)professional coordination/delegation |  | 1 | 1 | 1 | 1 | 1 | 1 |
| 30 | Multitasking |  |  |  | 1 |  | 1 |  |
| 31 | Orientation to new knowledge |  | 1 | 1 | 1 | 1 | 1 | 1 |
| 32 | Pattern recognition | 1 | 1 | 1 |  |  | 1 |  |
| 33 | planning approach to hypothesis testing |  | 1 | 1 |  | 1 |  | 1 |
| 34 | preparation | 1 | 1 | 1 | 1 |  | 1 | 1 |
| 35 | prioritization |  |  |  | 1 | 1 |  | 1 |
| 36 | professionalism |  |  |  | 1 |  | 1 | 1 |
| 37 | professional knowledge sharing | 1 | 1 |  | 1 |  |  | 1 |
| 38 | prompting patient | 1 |  | 1 |  | 1 | 1 |  |
| 39 | reflection | 1 | 1 | 1 |  |  |  |  |
| 40 | seeking out a second opinion/help-seeking | 1 |  | 1 | 1 | 1 | 1 | 1 |
| 41 | self-regulation |  | 1 | 1 | 1 |  |  | 1 |
| 42 | setting agenda |  | 1 |  | 1 | 1 | 1 |  |
| 43 | study | 1 | 1 | 1 | 1 | 1 | 1 | 1 |
| 44 | thoroughness | 1 | 1 | 1 |  | 1 | 1 | 1 |
| 45 | treatment planning | 1 |  | 1 | 1 |  | 1 | 1 |
| 46 | Uncertainty (e.g., comfort with or handling) |  | 1 | 1 | 1 | 1 | 1 | 1 |
| **Occurrences in all** | | **20** | **33** | **34** | **30** | **31** | **32** | **35** |
